# Winter storms accelerate the demise of sea ice in the Atlantic sector of the Arctic Ocean

**DOI:** 10.1038/s41598-019-45574-5

**Published:** 2019-06-25

**Authors:** Robert M. Graham, Polona Itkin, Amelie Meyer, Arild Sundfjord, Gunnar Spreen, Lars H. Smedsrud, Glen E. Liston, Bin Cheng, Lana Cohen, Dmitry Divine, Ilker Fer, Agneta Fransson, Sebastian Gerland, Jari Haapala, Stephen R. Hudson, A. Malin Johansson, Jennifer King, Ioanna Merkouriadi, Algot K. Peterson, Christine Provost, Achim Randelhoff, Annette Rinke, Anja Rösel, Nathalie Sennéchael, Von P. Walden, Pedro Duarte, Philipp Assmy, Harald Steen, Mats A. Granskog

**Affiliations:** 1 Norwegian Polar Institute, Fram Centre, Tromsø, Norway; 2 ARC Centre of Excellence for Climate Extremes, IMAS University of Tasmania, Hobart, Australia; 3 Institute of Environmental Physics, University of Bremen, Bergen, Germany; 4 Geophysical Institute, University of Bergen, Bergen, Norway; 5 Bjerknes Centre for Climate Research, Bergen, Norway; 6 Cooperative Institute for Research in the Atmosphere, Colorado State University, Fort Collins, Colorado, USA; 7 Finnish Meteorological Institute, Helsinki, Finland; 8 UiT The Arctic University of Norway, Tromsø, Norway; 9 Laboratoire LOCEAN-IPSL, Sorbonne Universités, UPMC, Univ. Paris 6, CNRS-IRD-MNHN, Paris, France; 10 Alfred Wegener Institute, Helmholtz Centre for Polar and Marine Research, Potsdam, Germany; 11 Washington State University, Department of Civil and Environmental Engineering, Pullman, Washington, USA

**Keywords:** Cryospheric science, Physical oceanography, Carbon cycle, Biooceanography, Atmospheric dynamics

## Abstract

A large retreat of sea-ice in the ‘stormy’ Atlantic Sector of the Arctic Ocean has become evident through a series of record minima for the winter maximum sea-ice extent since 2015. Results from the Norwegian young sea ICE (N-ICE2015) expedition, a five-month-long (Jan-Jun) drifting ice station in first and second year pack-ice north of Svalbard, showcase how sea-ice in this region is frequently affected by passing winter storms. Here we synthesise the interdisciplinary N-ICE2015 dataset, including independent observations of the atmosphere, snow, sea-ice, ocean, and ecosystem. We build upon recent results and illustrate the different mechanisms through which winter storms impact the coupled Arctic sea-ice system. These short-lived and episodic synoptic-scale events transport pulses of heat and moisture into the Arctic, which temporarily reduce radiative cooling and henceforth ice growth. Cumulative snowfall from each sequential storm deepens the snow pack and insulates the sea-ice, further inhibiting ice growth throughout the remaining winter season. Strong winds fracture the ice cover, enhance ocean-ice-atmosphere heat fluxes, and make the ice more susceptible to lateral melt. In conclusion, the legacy of Arctic winter storms for sea-ice and the ice-associated ecosystem in the Atlantic Sector lasts far beyond their short lifespan.

## Introduction

**Figure 1:**
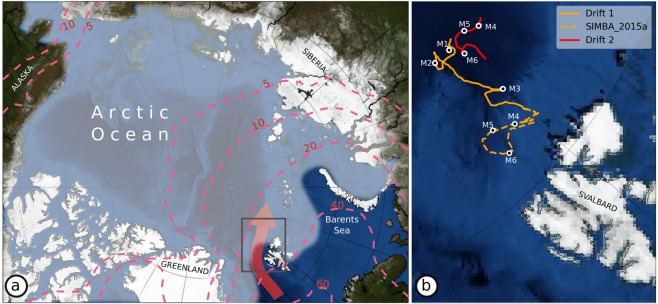
Schematic overview of the N-ICE2015 observations and Atlantic Water inflow pathway. (**a**) Schematic map of the Arctic Ocean with the mean 2015 winter sea ice extent in white shading and the approximate pathway of the Atlantic Water inflow shown by the red arrow. Dashed red contours indicate the climatological (1979–2015) mean number of extreme cyclone events^3^ per winter, based on ERA-Interim. Cyclone events are based on 6 hourly data, and so events lasting for one day correspond to four consecutive time steps. (**b**) Map showing the two winter drift trajectories of the N-ICE2015 camp (Drift 1 and Drift 2). Drift 1 covers the period 15 January – 21 February, and Drift 2 from 24 February – 19 March 2015. The drift of autonomous buoy SIMBA_2015a from 20 January – 16 March is also shown. The position of the N-ICE2015 camp and buoy at the onset of storms M1-M6 are highlighted by a white circles. The background images are from Blue Marble (©NASA).

The strongest storms in the Arctic Ocean typically occur during winter and originate from the North Atlantic Ocean^1,2^ (Fig. 1). The number and intensity of Arctic winter storms has increased over the period 1979–2016^3–5^. These storms often generate strong southerly winds that transport heat and moisture into the Arctic from the mid-latitudes, contributing to record breaking winter temperatures^6–9^. The increased heat and moisture further enhances downwelling longwave radiative fluxes and reduces surface heat loss, which decreases the potential for winter ice growth^4,5,10–13^. In addition, southerly winds push the ice-edge northwards and dynamically reduce the sea ice extent^8^.

Following the rise in winter storm activity^3^, the four lowest Arctic winter maximum sea ice extents in the satellite record were observed in the last four years (2015–2018)^14^. Similarly, four of the last five Arctic winters were among the warmest on record^9,12^. The most pronounced retreat of Arctic winter sea ice is in the Barents Sea, where sea ice has thinned^15^ and there has been a 47% reduction in ice extent over the satellite record^14^. The retreat of winter sea ice in the Barents Sea and north of Svalbard also coincides with the path of the warm Atlantic Water inflow^16,17^. This inflowing Atlantic Water has warmed and shoaled in recent years, and is likely a key mechanism of sea ice loss in the region^18–20^.

Many studies have investigated the impact of summer and winter storms on Arctic sea ice^21–25^. However, the full impact of winter storms on the Arctic remains unclear. *In-situ* measurements, of any type, from the central Arctic Ocean are scarce during winter. Therefore, earlier studies have relied heavily on atmospheric reanalyses, numerical models, remote sensing products, weather station data from the peripheries of the Arctic Ocean, or autonomous buoys. Such approaches present a number of problems. For example, reanalyses suffer from large temperature biases in the Arctic during winter, and there is a substantial spread in simulated precipitation products^26,27^. Uncertainties in snow depth on sea ice can induce large errors in ice thickness measurements from remote sensing^28–30^, and buoys reflect point observations in a system with large spatial variability over small distances^31,32^.

From January to June 2015 the Norwegian young sea-ICE (N-ICE2015) campaign took place in an area of relatively thin (<1.5 m) pack ice north of Svalbard^33^ (Fig. 1). N-ICE2015 was the most comprehensive field campaign to cover the winter season in the Arctic since the Surface HEat Budget of the Arctic (SHEBA) experiment in 1998, and provides detailed measurements of the atmosphere, ocean, sea ice, snow, and ecosystem^33^. N-ICE2015 is also the first winter campaign of such scale in the ‘stormy‘ Atlantic sector of the Arctic^3^. Most previous Arctic field campaigns took place on thick multi-year ice, whereas N-ICE2015 was situated in thinner and younger first and second year ice that now covers the majority of the Arctic Ocean. Several studies from N-ICE2015, including a special collection, were recently published for the individual components of the atmosphere-snow/ice-ocean-ecosystem^33^. The purpose of this study is to synthesise and build upon these recent results, to illustrate the multitude of ways in which winter storms impact the coupled sea-ice system in the Atlantic sector of the Arctic Ocean.

## Results

### The six N-ICE2015 winter storms

Cohen *et al*.^34^ documented how the N-ICE2015 camp observed six major winter storms (M1-M6), from 20 January and 21 March 2015 (Fig. 1). Here we analyse the tracks of these storms using the University of Melbourne’s cyclone identification scheme^35^ (Fig. S1). The six storms entered the Arctic Ocean from the North Atlantic through the Fram Strait, between Greenland and Svalbard^34^ (Fig. S1). Storms M1, M2, M4 and M6 continued northwards after passing through the Fram Strait and tracked northwest of the N-ICE2015 camp, but Storms M4 and M6 dissipated shortly after (Fig. S1). In contrast, Storms M3 and M5 followed a more eastward trajectory from Svalbard towards Franz Josef Land and the Barents and Kara Seas^34^. As a result, these storms initially passed south of the N-ICE2015 camp (Fig. S1). Storm M5 followed the most southerly trajectory, reaching a northernmost latitude of 78.3°N. All other storms tracked north of 84°N. Over the following sections, we summarise the common patterns and characteristics of these six Arctic winter storms. However, we note that the overall impact of an individual storm is unique and will be dependent on the storm’s track, strength and lifespan.

### Winter storms transport heat and moisture into the Arctic

**Figure 2:**
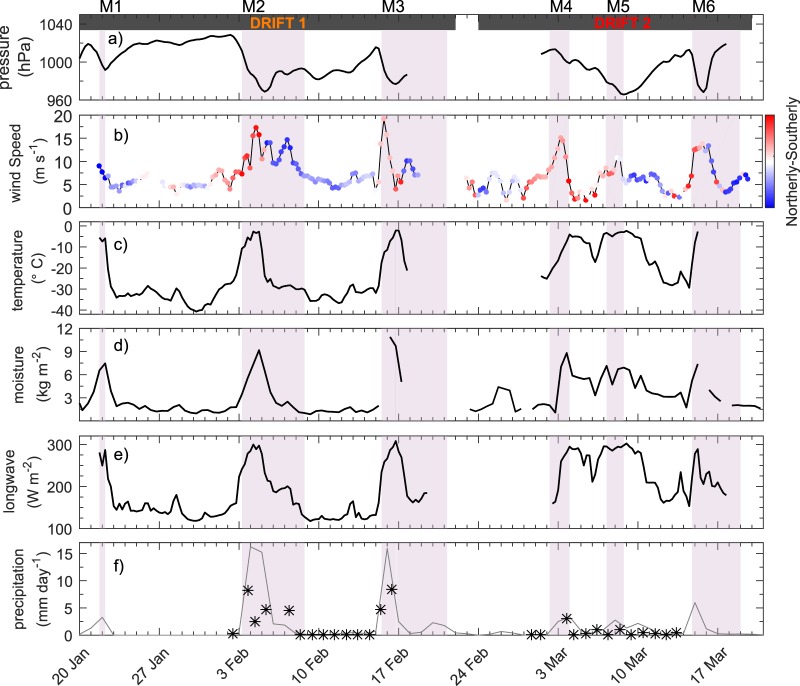
Time series of meteorological observations during the first two N-ICE2015 ice drifts in January–March 2015. Major storm periods (M1-M6) at the ice camp are shaded in purple^34^. (**a**) Mean sea level pressure^34^, (**b**) 10 m wind speed and direction^34^, (**c**) 2 m air temperature^34^, (**d**) vertically integrated atmospheric moisture content^36^, (**e**) downwelling longwave radiative flux at surface^37^ (positive downwards i.e. into the ice), and f) daily precipitation^34^, observations shown by black stars and grey line shows data from ERA-Interim^83^. Timing of the two drifts of ice camps (see Fig. 1) are indicated by the black bars on top of panel (a). Figure shows observations first presented by Cohen *et al*.^34^, Kayser *et al*.^36^, and Walden *et al*.^37^.

**Figure 3:**
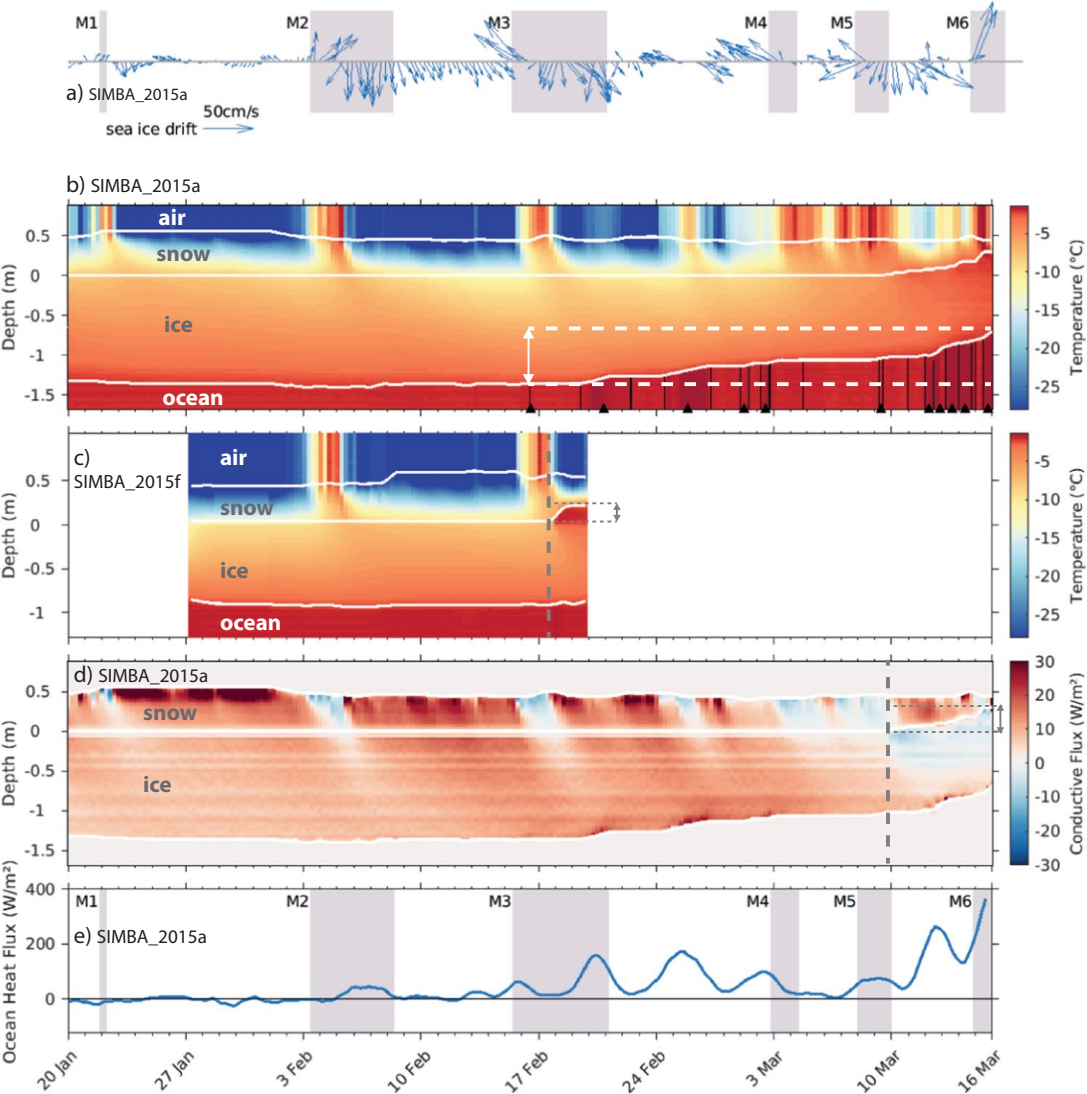
Time-series of ice mass balance buoy (SIMBA_2015a & SIMBA_2015f) data during and between the six storms, M1-M6. (**a**) Sea ice drift speed and direction. (**b**,**c**) Temperature profile through (top to bottom) air, snow, ice, and surface ocean. All interfaces are delineated by solid white lines. The bottom melt rate of about 70 cm/month in panel (b) is marked by white dashed lines and the double-headed arrow. Seawater temperatures higher than −1.8 °C are delineated by black contours. The detection of such warm ocean water is additionally marked by black triangles at the bottom of the plot. (**d**) Conductive heat fluxes through snow and ice for SIMBA_2015a. Upward directed fluxes have positive values and downwards negative. Vertical dashed grey line indicates timing of flooding at the snow-ice interface and the start of the snow-ice formation. The grey thin dashed lines and the double-headed arrow mark the thickness of the flooded/snow-ice layer of about 25 cm. Note that values at the ice/snow interface are not used for clarity; do to some spurious values after deployment. (**e**) Inferred ocean heat flux at the ice-ocean interface (positive upwards i.e. into the ice) from SIMBA_2015a. The storm periods (M1-M6) are shaded as in Fig. 2. Figure shows observations first presented by Provost *et al*.^38^.

Due to the cyclonic circulation of storms, the six storms were found to be characterized by two distinct phases as they tracked north-eastwards over the camp^34^ (Fig. 2). The first phase of each storm corresponds to the time period before the centre of the storm reached the N-ICE2015 camp (Figs 2a, S2). Kayser *et al*.^36^ showed that this phase was associated with strong winds from the south that transport warm and moist air into the central Arctic Ocean, from the North Atlantic (Figs 2b–d, S2). The transport of heat and moisture contributed to cloud formation, and resulted in an abrupt increase in downward longwave radiation^37^ (Fig. 2e). The atmospheric surface energy budget subsequently transitioned from net heat loss to near-equilibrium^37^. The advection of warm and moist air caused the temperature and humidity to increase rapidly throughout the full height of the atmosphere^36^. On four occasions, near-surface air temperatures rose by more than 20 °C in 48 hours^5^ (Fig. 2c). During this phase of each winter storm, the atmospheric boundary layer temperature was warmer than the surface snow and sea ice (Fig. 3). This triggered downward conductive heat fluxes that warmed the snow and upper layers of the ice, and inhibited thermodynamic ice growth^38^ (Fig. 3d). However, near-surface air temperatures never exceeded 0 °C, and no surface melt was observed (Fig. 2c).

The second phase of each storm corresponds to the time after the low pressure passed over the camp^34^ (Figs 2a, S2). During this phase, the wind direction typically changed from southerly to northerly^34,36^ **(**Fig. 2b**)**. The northerly winds advected cold and dry, polar air, over the camp^36^ (Fig. 2b–d). The strength of this cold air advection and rate of cooling were dependent on the track and lifespan of the storms^34^. Storms that followed a trajectory northwards into the central Arctic Ocean were found to result in the strongest cooling, due to northwesterly winds that advected very cold and dry air from the north coast of Greenland towards the camp^36^ (Fig. S2). In some cases near surface air temperatures dropped from near 0 °C to below −30 °C in 24 hours^5^ (Fig. 2c). In contrast, the second phase of storm M5, which followed a more southern trajectory from Svalbard towards Franz Josef Land, was associated with easterly winds that advected comparatively warmer air from the Barents and Kara Seas toward the camp, and resulted in a slower cooling rate^34^ (Figs 2, S2). Following each storms’ passage, colder and drier conditions prevailed, with clear skies, low wind speeds and strong radiative cooling^5,34^ (Fig. 2).

Graham *et al*.^5^ showed that the sequence of events documented during the N-ICE2015 winter storms was similar to those observed during the 1998 SHEBA experiment in the Beaufort Sea^37,39,40^. However, the N-ICE2015 storms were more frequent and stronger, with lower surface pressures, higher wind speeds, higher air temperatures, and more precipitation^5^ (Fig. 2). This is consistent with the positive trend in the number and intensity of Arctic winter storms from 1979–2015^3^. However, differences between the N-ICE2015 and SHEBA storms are primarily due to the proximity of N-ICE2015 to the open ocean and active North Atlantic storm track^2,3,5,11^. In contrast, SHEBA was situated further in the central Arctic Ocean, such that storms originating from either the Atlantic or Pacific Sectors would have weakened considerably before reaching the camp^39^.

### Snow from autumn and winter storms limits ice growth

The thermal conductivity of snow is an order of magnitude lower than that of sea ice^41^, and therefore snow effectively insulates the ocean from the cold atmosphere and reduces sea ice growth^29,41^. At the start of the N-ICE2015 field campaign, a 0.4–0.6 m deep snowpack was observed on first and second year ice floes^38,42,43^ (Fig. 3b,c). This is double the widely used snow-on-sea-ice climatology for January in this region^44^. The disparity in snow depth from the climatology likely reflects the lack of representative early winter observations from this region^28,42–44^. However, it is also possible that the unexpectedly deep snow pack is the result of more frequent and more intense winter storms bringing more precipitation to this region in recent years^3,45^. All snowfall greater than trace amounts during the N-ICE2015 winter was associated with a storm^34^ (Fig. 2f). Studies analysing the structure of the N-ICE2015 snowpack and atmospheric reanalyses, concluded that the snowpack deepened during a series of storms in late autumn and early winter^32,46^.

The extensive N-ICE2015 observations revealed minimal (<0.1 m) thermodynamic growth on the underside of snow-covered first and second year sea ice between January and March 2015, despite prolonged periods with air temperatures below −30 °C^38,42^ (Fig. 3b,c). In contrast, comparably low air temperatures during SHEBA contributed to substantial (0.2 m) winter sea ice growth when the snow pack was only 0.25 m deep, even though the ice was more than 0.5 m thicker compared to N-ICE2015^31,39^. However, ice growth rates are also controlled by many factors other than snow depth^12^.

**Figure 4:**
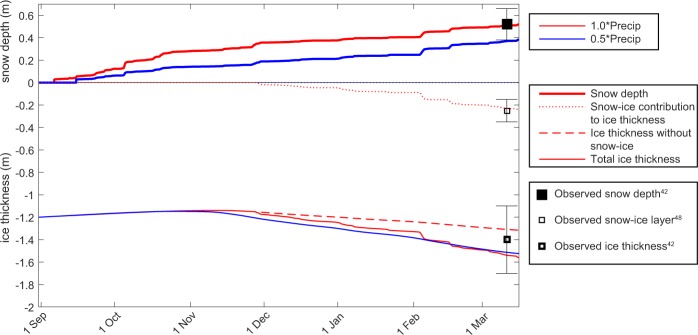
Evolution of snow and ice during the N-ICE2015 winter and preceding autumn, based on single column numerical model (HIGHTSI^90^). The model is forced by N-ICE2015 observations and ERA-Interim reanalyses^83^. Two simulations explore the impact of changes in precipitation: 1*Precip (red, Total precipitation from ERA-Interim), 0.5*Precip (blue, 50% total precipitation from ERA-Interim). The simulations are compared to observed sea ice thickness, snow depth^42^ and snow-ice layer thickness^48^ on second year ice during Drift 1 of N-ICE2015. Squares and whiskers depict mean and standard deviation.

Here we isolate the impact of snow insulation on ice growth rates during the N-ICE2015 winter using a 1-D sea ice model (Methods). Specifically, we perform two simulations. In the first simulation, we force the model with the total precipitation from an atmospheric reanalysis. This produces a 0.4 m snowpack in January, which is consistent with N-ICE2015 observations^43,46^ (Fig. 4). The total thermodynamic ice growth for this multi-year ice floe is approximately 0.10 m over the entire winter season (Fig. 4). For the second simulation, we reduce the total precipitation by a factor of two, but otherwise keep the model setup identical. The lower precipitation produces a 0.25 m snowpack in January that is more consistent with the Warren *et al*.^44^ climatology for the region. With this thinner snowpack, thermodynamic winter ice growth increases to approximately 0.35 m (Fig. 4). Hence, these simulations indicate that the insulation provided by the deep snowpack during N-ICE2015 likely reduced thermodynamic winter ice growth by 0.25 m, relative to a climatological snow depth^44^.

Several recent studies have highlighted the role of increased atmospheric heat and moisture transport, by more frequent storms, for reducing winter sea ice growth in the Atlantic Sector of the Arctic Ocean^4,10,11,13,39^. However, the extreme transient warming events during N-ICE2015 winter storms had limited impact on growth rates of snow covered sea ice (Fig. 3). Instead, we emphasise that more frequent winter storms likely contribute to more precipitation and a deeper snowpack. Our model simulations and the *in-situ* winter observations from N-ICE2015 demonstrate that the additional insulation provided by a deep snowpack can have a larger impact on winter ice growth than any transient atmospheric warming during storms. This is because a deep snowpack effectively decouples the ice from the atmosphere. Downward directed conductive heat fluxes propagated by warm air during storms dissipate before they reach the ice-water interface and likewise, snow insulation prevents effective upward directed fluxes to cool the ocean surface during cold quiescent period between the storms, resulting in minimal ice growth rates (Fig. 3d). While the crucial role of snow insulation on winter ice growth is well known^31,41,47^, the consequences of snow deposited on sea ice during winter storms for Arctic sea ice have largely been overlooked. This is due to a lack of *in-situ* observations and large uncertainties in precipitation and snow depths from models, reanalyses, and remote sensing products^29^. Interestingly, we note here that the impact of snow insulation on ice growth is known to be largest for snow falling on thin sea ice early in the winter season^46^, and the main increase in winter storm activity from 1979–2016 is reported during the early winter^3^.

### Winter storms promote snow-ice formation

While thermodynamic ice growth was limited beneath the deep snow pack, an important mechanism of ice growth during the N-ICE2015 winter was shown to be snow-ice formation^38,46,48^ (Fig. 3). Snow-ice formation requires negative freeboard, caused by a heavy snow load that pushes the ice surface below sea level. In addition, a pathway is required for seawater to flood the ice surface (Fig. S3). Snow-ice formation is common for Antarctic sea ice where the seasonal ice cover is relatively thin and there is ample snowfall, but is not considered typical for the comparatively dry Arctic^49^.

In the Atlantic Sector of the Arctic Ocean, frequent storms can contribute sufficient snowfall to induce negative freeboard. Rösel *et al*.^42^ observed extensive areas of negative freeboard during N-ICE2015. Large areas with negative freeboard remained dry with no observed flooding^42^, but several autonomous buoys detected snow flooding shortly after strong sea ice deformation events during certain storms^38^ (Fig. 3b,c). Seawater likely flooded the snow through cracks formed during sea ice convergence, triggered by storms. These flooded snow layers began to freeze and form snow-ice during the second phase of the storm, when cold and dry air was advected from the north^38^. Sea-ice cores from N-ICE2015 revealed saline layers of snow-ice of up to 0.3 m thick, demonstrating for the first time that snow-ice contributed significantly to the sea ice mass balance of Arctic pack ice^48^.

Here we explore the impact of changing winter precipitation on snow-ice formation, using our simulations with the 1-D sea ice model. When forced with the total precipitation from an atmospheric reanalysis, the model simulates a 0.24 m thick layer of snow-ice for the N-ICE2015 winter (Fig. 4). This is in agreement with ice core anlayses^48^. In contrast, when the total precipitation is reduced by 50%, the simulated snow load is insufficient to cause negative freeboard and no snow-ice forms in the model (Fig. 4). These simulations indicate that the potential snow-ice contribution (0.24 m) during N-ICE2015 could approximately compensate for the reduced thermodynamic growth from snow insulation (0.25 m), relative to a case with climatological snow depth. However, this model assumes that all areas with negative freeboard are flooded, which we know is not truly representative because large areas with negative freeboard were never flooded during N-ICE2015^42^. Hence, the total snow-ice contribution to ice mass balance is unlikely to reach its full potential and fully compensate the effects of snow insulation.

We note that the presence of saline snow and also the internal structure of the snow pack (e.g. ice crusts observed during N-ICE2015^43^) can cause overestimation of sea ice thickness by space-borne radar altimeters such as CryoSat-2^30,50^. King *et al*.^30^ showed that radar reflections from CryoSat-2 in the N-ICE2015 region during spring 2015 were closer to the snow freeboard than ice freeboard, and resulted in a systematic overestimation of sea-ice thickness by a factor of two. This highlights the need to give careful consideration of snow properties when interpreting satellite radar altimetry data in this region.

We speculate that continued thinning of Arctic sea ice may increase the potential for future flooding and snow-ice formation in the Atlantic Sector of the Arctic Ocean. Nonetheless, historic and projected trends in Arctic precipitation are highly uncertain^27,51^. With large regional differences across the Arctic, the net effect of thinning ice and changes in precipitation remain unknown. Later freeze-onsets and/or a trend towards more liquid precipitation (i.e. rain) could instead act to reduce the future snow load on sea ice and decrease the potential for snow-ice formation^14,27,52,53^.

### Winter storms open leads enabling new ice growth

**Figure 5:**
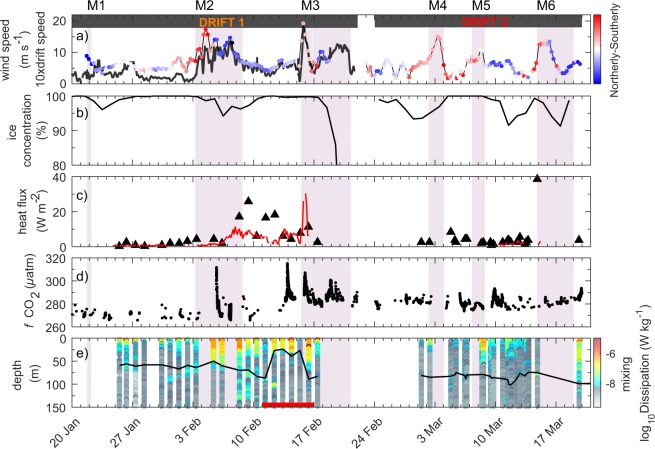
Time series of atmospheric, sea-ice, and oceanographic observations during the first two N-ICE2015 ice drifts in January–March 2015. (**a**) 10 m wind speed and direction^34^ (coloured dots), and mean sea ice drift speed^54^ from GPS buoy array (thick black line). (**b**) Sea ice concentration at position of the ship from AMSR-2^57^ (the steep drop off at the end of Drift 1 is associated with entering the marginal ice zone). (**c**) The ocean heat flux (positive upward) measured at the ice-ocean interface is given by red line^59^. Ocean heat fluxes measured at the depth of the pycnocline are given by black triangles^60^. (**d**) Fugacity of CO_2_ (*f*CO_2_, µatm) measured in sea water at 5 m depth^57^. (**e**) Ocean mixing given by dissipation rate^60^ (colour bar, warm colours correspond to stronger mixing), mixed layer depth^62^ (black line), and presence of Atlantic Water (red bar). Bold red bar indicates Atlantic Water (>2 °C) shallower than 250 m^62^. The two drift periods are highlighted by black bars in top of panel (a). Storm periods (M1-M6) are shaded as in Fig. 2. Figure shows observations first presented by Itkin *et al*.^54^, Meyer *et al*.^60,62^, Peterson *et al*.^59^ and Fransson *et al*.^57^.

Storm-driven ice deformation events are important for creating leads. During the first phase of each N-ICE2015 winter storm, southerly winds pushed the ice northwards into the ice pack^54^. This compacted the ice pack and increased the ice concentration around the N-ICE2015 camp (Figs 3a and 5a,b). This deformation was shown to predominantly occur along refrozen leads where the ice was weakest, but the compression and shear also cracked thicker snow-covered ice floes^55^. Due to this compression, the ratio of ice drift speed to wind speed was found to be lower during the first phase of each storm^54,55^.

**Figure 6:**
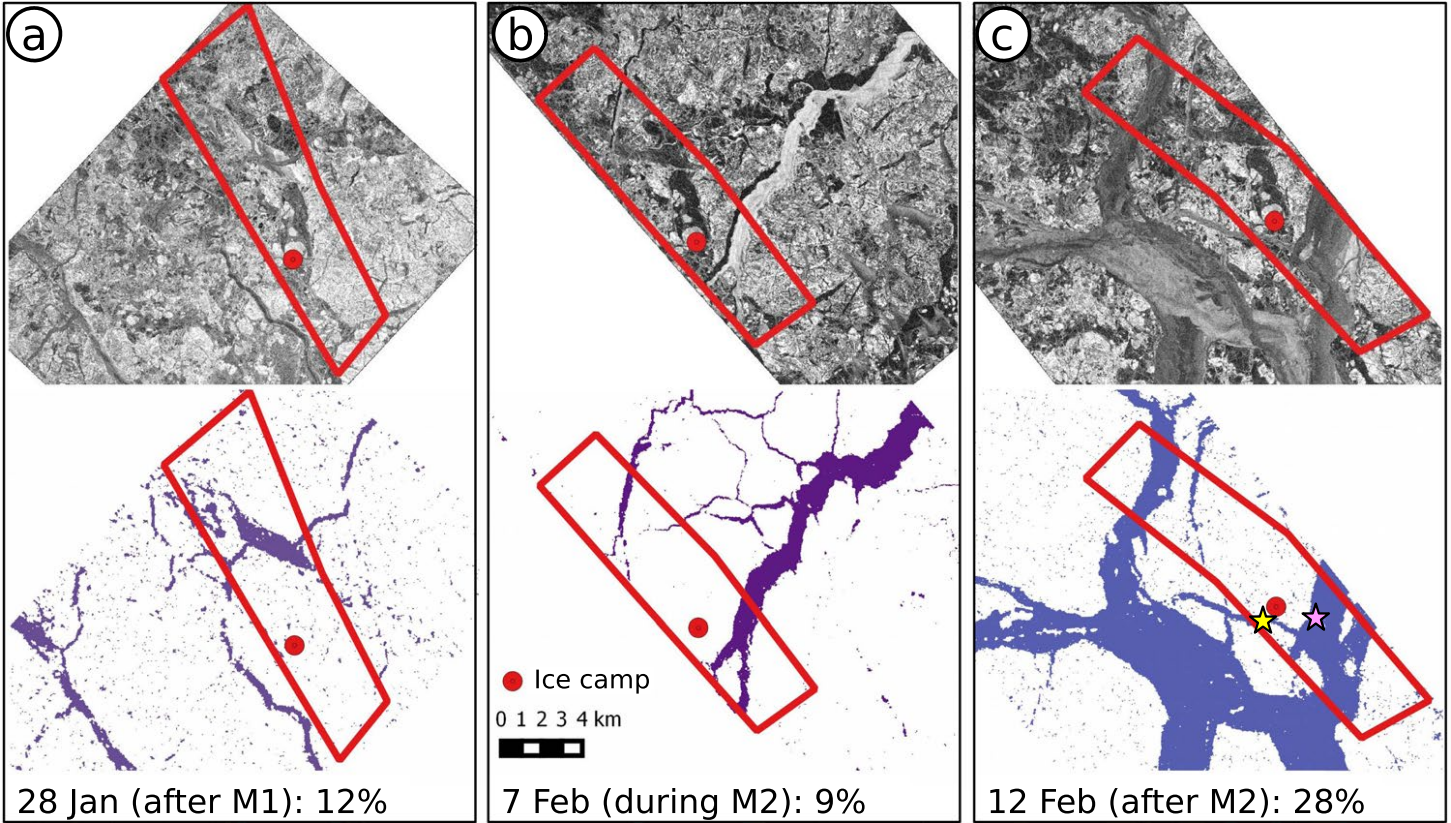
Opening of leads following Storm M2. TerraSAR-X (TSX, © DLR 2015) synthetic aperture radar (SAR) images of the sea ice cover (top) surrounding the N-ICE2015 ice camp on (**a**) 28 January, (**b**) 7 February and (**c**) 12 February 2015. On bottom of each panel SAR images are classified into leads (open water and thin ice, purple/blue shades) and the rest of the ice (white). The red polygon on the images designates approx. 70 km^2^ between corner points that were manually tracked from image to image. The lead fraction within the polygon is given at the bottom of each plot. The locations where ice thickness in leads was measured on February 12 and 14 are marked by yellow and pink stars on panel (c). See Methods for full details on image processing and field measurements.

During the second phase of each storm, northerly winds transported the sea ice southwards, towards the ice edge, promoting free drift^54,55^ (Fig. 3a). Without resistance from the ice pack, the southward ice drift was found to be more rapid and generated strong divergence, opening new leads in the broken ice^54,55^ (Fig. 6). These processes can be seen by the drop in sea ice concentration around the camp, during the second phase storms M1-M3, and M5-M6 (Fig. 5b). Similarly, following the second phase of storm M2 we see a large increase in the ratio of sea ice drift speeds to wind speeds^54^ (Fig. 5a). The combination of open leads (i.e. lower ice concentration) and strong winds likely enabled large fluxes of heat, moisture, and gases between the ocean and atmosphere at these times^56,57^. For example, Fransson *et al*.^57^ estimated that the CO_2_ flux from the atmosphere into the ocean was approximately 20 times higher during storm periods, compared with average wind speed conditions and fewer open leads^57^.

Here we use high resolution satellite imagery to show the changing lead fraction in the vicinity of the N-ICE2015 camp, before (12%), during (9%), and after (28%) storm M2 (Methods, Fig. 6). Following the passage of each storm, any newly opened leads rapidly froze after cold and dry conditions returned, creating extensive areas of new ice. *In-situ* measurements from new ice forming in the leads opened by storm M2, revealed an ice thickness of 0.28 m after five days (Fig. 6c). We emphasise here that this new ice growth in leads would not have occurred without the aid of the storm, because growth rates under the existing snow-covered sea ice were negligible^38,42,46^ (Fig. 3b,c). By assuming an average ice thicknesses of 0.28 m in newly opened leads, and 1.0–1.5 m for existing snow covered ice^42^, we estimate that storms M1 and M2 may have contributed each a 2–10% increase of sea ice volume in the vicinity of the N-ICE2015 camp (Fig. 6). However, the net effect of a storm on sea ice volume is complex, and will depend on local ice and ocean conditions as well as the path, size and strength of the storm.

### Winter storms enhance ocean mixing, heat fluxes, and ice melt

Sea ice dampens energy transfers between the atmosphere and ocean, and therefore the Arctic Ocean is traditionally considered to be energetically ‘quiet’ with weak turbulent mixing^58^. However, strong winds during the N-ICE2015 winter storms enhanced ice drift speeds^54^ (Figs 3a and 5a), which increased ocean-ice velocity shear^59^. These processes were found to generate mixing in the upper ocean, and led to increased transfer of ocean heat towards the ice^59,60^ (Fig. 5c–e). Observed winter ocean-ice heat fluxes typically more than tripled from 2 W m^−2^ to 7 W m^−2^ during storm periods (Fig. 5c, Methods), further impeding ice growth and in several cases initiating bottom melt^59,60^ (Fig. 3b).

Ocean mixing is particularly important in many regions of the Arctic Ocean because warm water of Atlantic origin is located below cold and fresh Polar Surface Water^61^. Along the continental slope north of Svalbard, warm Atlantic Water (>2 °C) is found close to the surface (Figs 1, 5e). Vertical mixing thus generates enhanced ocean heat fluxes. The magnitude of this heat flux is dependent on the mixing rate, as well as the depth and temperature of the warm water. During the N-ICE2015 winter drift over the deep Nansen Basin, Modified Atlantic Water (>0 °C) was found at approximately 100 m depth^62^. Under calm conditions in the deep Nansen Basin, Meyer *et al*.^60^ observed ocean heat fluxes at the pycnocline of approximately 3 W m^−2^ (Fig. S1). However, during storm periods, wind-driven mixing almost doubled the pycnocline heat fluxes to 5.5 W m^−2^ (Methods, Fig. S4a). These enhanced ocean heat fluxes are relatively small in comparison to changes in the atmospheric surface energy budget during storms^37^ and were insufficient to induce ice bottom melt, but nevertheless acted to further suppress ice growth (Figs 2e and 5c). Previous work using autonomous buoy measurements have inferred enhanced ice-ocean heat fluxes during winter storms in the Beaufort Sea^25^. It is therefore expected that these conditions in the Nansen Basin are representative of large areas of the central Arctic Ocean.

During storm M3, the N-ICE2015 camp drifted over the Atlantic Water inflow path along the Svalbard continental shelf. Here, Peterson *et al*.^59^ observed ocean-ice heat fluxes of 30 W m^−2^, resulting in substantial ice bottom melt (Figs 3b and 5c–e). After the onset of bottom melt, the ice floe began to break-up and measurements were stopped to safely recover instruments. Larger heat fluxes would likely have been recorded if measurements had continued. Provost *et al*.^38^ inferred ocean-ice heat fluxes from several buoys located near the N-ICE2015 camp at this time, which were on the order of 100–200 W m^−2^ (Fig. 3e). Similarly, during storm M6, a buoy located over the Atlantic Water inflow path experienced ice bottom melt rates of 25 cm day^−1^, equivalent to an ocean-ice heat flux of 400 W m^−2^ (Fig. 3). An ocean mooring array in this region previously recorded episodic vertical ocean heat fluxes in excess of 100 W m^−2^, during the winter of 2012–2013^63^, suggesting that ocean heat fluxes of this magnitude are not uncommon here. On both occasions that the N-ICE2015 camp drifted over the Atlantic Water inflow in winter, a storm was ongoing resulting in ocean-ice heat fluxes of 30 W m^−2^ due to the combined impact of storms with the presence of Atlantic Water at shallow depth (Fig. S4b). The timing of the storms also meant that winter-time ocean heat flux measurements over the Atlantic Water inflow are only available for stormy conditions, and not quiescent periods^59,60^. Due to strong tidal mixing in this region, we expect ocean heat fluxes over the Atlantic Water inflow path to be large even during quiescent conditions^19,64,65^. Nonetheless, spring and summer ocean heat flux measurements from N-ICE2015, and winter-time buoy measurements indicate a clear additional effect from storm driven mixing^38,59,60^.

**Figure 7:**
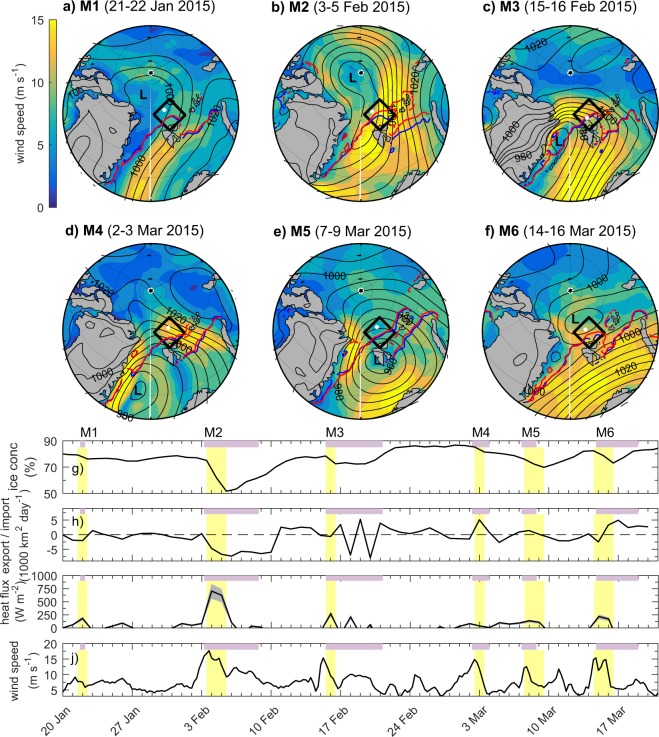
Large scale impact of storms. Maps of 10 m wind speed (colour) and mean sea level pressure field (black contours, hPa) averaged over the period of the six major N-ICE2015 winter storms (**a**–**f**), from ERA-Interim^83^. Winds blow anti-clockwise around the storm’s low pressure centre (L). The 85% OSI-SAF sea ice concentration is indicated before and after the storm by the blue and red lines respectively. The position of the N-ICE2015 camp at the onset of each storm is given by a white dot. g) Time series of mean sea ice concentration in the Atlantic Water inflow box north of Svalbard. (**h**) Net import (+)/export (−) of sea ice area to/from the Atlantic Water inflow box, based on OSI-SAF sea ice drift data. (**i**) Ocean heat flux required to melt areal loss of sea ice (accounting for import/export) assuming a mean thickness ranging from 1.0–1.5 m (grey shaded area, black line corresponds to 1.25 m). (**j**) Mean 10 m wind speed in the Atlantic Water inflow box from ERA-Interim. Storm periods (M1-M6) observed at N-ICE2015 camp are highlighted in purple. Time periods for averaging in panels (a–f) correspond to the periods shaded in yellow. See Methods for further details.

**Figure 8:**
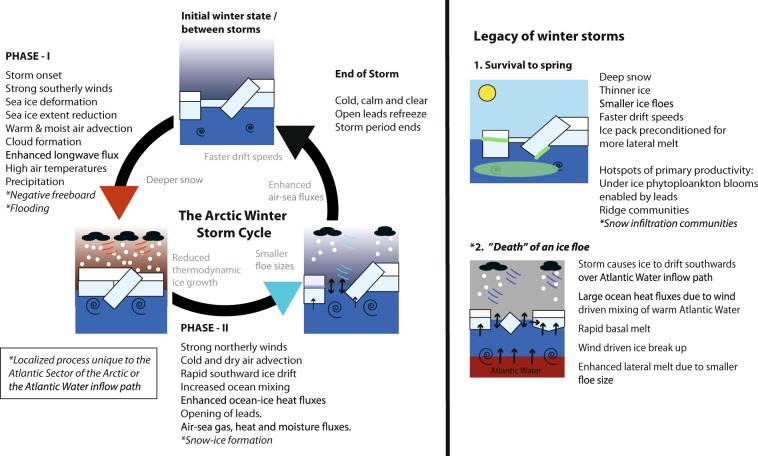
Schematic of processes related to Arctic winter storms. In the first storm phase, strong southerly winds compress the ice cover and transport atmospheric heat, moisture, and precipitation into the Arctic. The atmospheric warming temporarily slows ice growth, and snow accumulation effectively inhibits bottom ice growth throughout the remaining winter season. If the snow load is sufficient to induce negative freeboard, snow may be flooded along the cracks in the ice during a storm. This flooded snow may then refreeze, forming snow-ice. In the second phase, northerly winds transport ice southwards towards an unconstrained ice edge. The rapid drift opens numerous leads, allowing intensive air-ocean gas and heat exchange and strong upper-ocean mixing, resulting in enhanced ocean heat fluxes. Along the Atlantic Water inflow path, storms mix up heat from warm subsurface waters and effectively melt large volumes of ice even in winter. After the storm has passed, cold and calm conditions return, allowing new ice to grow in leads. When the next winter storm arrives, it further drives the ice cover into a relatively thin-ice, snow-covered mosaic of strongly deformed ice floes that also impacts ice-associated ecosystems by shaping habitats and light conditions.

We now investigate the large-scale impact of ocean-ice heat fluxes from storm-driven mixing over the Atlantic Water inflow path, using satellite observations of ice concentration and drift speeds (Methods). We find that each N-ICE2015 winter storm is associated with a reduction in sea-ice concentration of up to 25%, over the Atlantic Water inflow path north of Svalbard (Fig. 7a–g). After ice advection is taken into account, we estimate that an ocean heat flux of up to 600 W m^−2^ would be required to fully melt the sea-ice area lost during storm M2 (Fig. 7g–i). During other storms, required heat fluxes range from 200–300 W m^−2^. These values are of a similar order of magnitude as ocean-ice heat fluxes inferred from buoys located over the Atlantic Water inflow during N-ICE2015 winter storms^38^ (Fig. 3e). Sea-ice concentrations typically return to pre-storm levels within a week of a storm (Fig. 7g,j), presumably after the reduction in wind speeds, ocean mixing, and ocean-ice heat fluxes allows ice growth to resume in open water areas and leads (Fig. 5–8). However, the rapid succession of storms during the N-ICE2015 winter causes repeated ice loss in this region. Ivanov *et al*.^18^ have previously shown that in winters with frequent and severe storms, like 2015, the sea ice concentration north of Svalbard remained low for several weeks, and in extreme cases open water extended along the path of the Atlantic Water inflow as far east as Franz-Josef Land. The N-ICE2015 observations demonstrate that storm driven ocean mixing provides an effective mechanism for releasing heat from the warm Atlantic Water inflow and melting sea ice during winter^59,60^.

## Discussion

### Legacy of winter storms for the arctic ecosystem

The spike in surface ocean CO_2_ concentrations observed at the N-ICE2015 camp during and after storms M2 and M3, signals deeper CO_2_ and nutrient rich water being mixed upwards^57^ (Fig. 5d). Storm-driven ocean mixing during N-ICE2015 was found to enhance nitrate fluxes into the mixed layer, contributing to winter-restocking of nutrients for spring biological activity^66,67^.

The legacy of the six N-ICE2015 winter storms was an ice-pack formed of small floes covered by thick snow, and interconnected by numerous open or refrozen leads with very thin snow cover^68^ (Figs 3 and 6**)**. It is commonly understood that the spring algae bloom can only develop after the light-attenuating snow layer melts^69^. However, late spring observations from N-ICE2015, made in the same region but over different ice floes, showed that the increased light transmission through the numerous leads was sufficient for the development of an early under-ice phytoplankton bloom during the pre-melt season^70–72^ (Fig. 8).

Pressure ridges created by deformation during winter storms were identified as another biological hotspot during the N-ICE2015 spring^73^ (Fig. 8). The keels of ridges, especially those facing adjacent leads, provide a sheltered and favourable growth environment for ice algae^73^. Localised flooding caused by the heavy snow load and negative freeboard in spring was found to allow the infiltration of phytoplankton from the surface ocean to the snow-ice interface^73^ (Fig. 8). These snow-infiltration communities are common in the Antarctic seasonal ice cover, but few reports exist in the Arctic^74,75^. As with snow-ice formation, we speculate that this phenomenon may become more common in the Atlantic Sector of the Arctic Ocean, if sea ice continues to thin and/or snowfall increases in the winter and spring^45,73,76^.

### Storms precondition ice for faster summer melt

Our findings show that sea ice deformation and divergence during storms can promote new ice growth in leads, and may locally increase the sea ice volume. However, deformed ice is known to be more susceptible to melt^24^. Itkin *et al*.^54^ recorded sea ice deformation rates around the N-ICE2015 camp that were 10% higher than those measured in other parts of the Arctic Ocean. Sea ice deformation breaks the ice into smaller floes that drift more easily with the wind^77^. Following storm M2, a 60% step increase in the ratio of ice drift speed to wind speed was observed^54^ (Fig. 5a). The increased drift speeds, following storm driven sea ice deformation, will likely accelerate the transport of older and thicker sea ice out of the central Arctic and into the marginal ice zone of the Atlantic Water inflow path (Fig. 3a). In addition, smaller floe sizes following storms will precondition the ice for more lateral melt^24^. We further speculate that winter storms may contribute to an increased lead fraction in summer, reducing the albedo of the wider region.

It is unclear how a deep winter snow pack, resulting from frequent winter storms, would ultimately influence the summer melt season. This could postpone the initial melt pond development at the snow-ice interface, resulting in a higher albedo and thus slowing the melt onset. Fresh snowfall from any spring storms would also increase the surface albedo and have a large influence on the melt onset^78^. After melt onset, we might expect a deep snow pack to result in a large melt pond fraction with low albedo, which would accelerate melt^79^. However, these melt ponds may drain rapidly, due to the heavily fractured ice floes. The N-ICE2015 campaign ended due to floe break up prior to the widespread development of melt ponds, and so it was not possible to study these processes. We highlight this as an important period to target for future field campaigns.

## Conclusions

In this study we synthesise and build upon interdisciplinary results from N-ICE2015, to illustrate the multitude of ways in which winter storms accelerate the demise of sea ice in the Atlantic Sector of the Arctic Ocean (Fig. 8). Winter storms transport pulses of heat and moisture into the Arctic atmosphere, which temporarily impede ice growth. However, ice-growth does not resume after these short-lived warming events. Snow deposited during storms insulates the ice from the cold atmosphere and reduces ice growth throughout the entire winter season. Strong winds break the ice into smaller floes that drift faster and will experience more lateral melt. Moreover, northerly winds, in the latter phase of storms, accelerate the transport of thicker-older ice in the Transpolar Drift towards the warm Atlantic Water inflow. Over the Atlantic Water inflow path, storm-driven mixing can generate large ocean heat fluxes that melt the ice from below, even in mid-winter. Hence, the cumulative effect of frequent winter storms in the Atlantic Sector of the Arctic Ocean is a thinner, weaker, and younger snow-laden ice-pack (Fig. 8).

The impact of an individual storm on sea ice is complex, and will be highly dependent on the timing, track, strength and lifespan of a storm. We conclude that storms which occur early in the winter (i.e. shortly after freeze-up) are likely to have a more negative impact on winter ice growth, compared with storms later in the season. This is due to a combination of the lasting effects of snow insulation, and the fact thinner sea ice is more sensitive to the effects of snow insulation than older thicker ice^29,41,46,47^. In contrast, storms that occur later in the winter season, after a deep snow pack is established, may promote ice growth by dynamically opening leads where new ice growth can take place. Additionally, in cases when the snow depth is sufficient to induce negative freeboard, winter storms may trigger flooding and snow-ice formation.

While the observations presented in this paper are from storms in the Atlantic Sector, many processes we describe will be applicable for winter storms in other regions of the Arctic Ocean (Fig. 8). Specifically, the effects of snow-insulation, sea-ice deformation, and reduced radiative cooling, are likely to be important over the entire Arctic Ocean. We expect storm driven ice-ocean heat fluxes on the order of 5 W m^−2^ to be widespread over the Arctic Ocean^25^. These heat fluxes are unlikely to cause bottom melt, but will reduce winter ice growth. Large ocean heat fluxes capable of causing rapid bottom melt are a regional phenomenon unique to the Atlantic Water inflow path. Similarly, we only expect snow-ice formation to occur in the Atlantic Sector of the Arctic Ocean, where winter precipitation totals are sufficiently high^27^ relative to the thinning sea ice to make it susceptible to flooding^15,42^.

The N-ICE2015 observations from the Atlantic Sector of the Arctic Ocean are especially important because this region experiences the most frequent and strongest winter storms^3^. While winter storm activity has increased here in recent years, it is unclear whether this trend will continue into the future^3,80^. Nevertheless, the overall impact of winter storms on sea ice will likely increase in a warmer Arctic. With a thinner sea-ice cover^76^ and warmer and shallower Atlantic Water^19^, the tight air-snow-ice-ocean coupling we observed during storms will strengthen. Therefore, we expect more break-ups and ridging, more leads, greater ocean heat fluxes, faster melt, and possibly more flooding in response to winter storms. These emerging physical properties of the new Arctic sea ice regime will also have consequences for the ice-associated ecosystem.

The spatial scales of many processes highlighted above are typically small (<1000 m). This hampers the detection of these processes from remote sensing and ultimately their representation in climate models. *In-situ* observations are thus essential to improve our understanding of these processes. We emphasize that the autumn-freeze up and winter season are important periods to target in future field campaigns as it sets the scene for the summer melt period.

## Methods

### Overview of field campaign

The Norwegian young sea ice expedition (N-ICE2015) took place in the sea ice pack north of Svalbard from January – June 2015, onboard the Research Vessel Lance (Fig. 1). The ship was moored to an ice floe and a drifting ice camp was established at approximately 83°N. Due to rapid ice drift southwards to the marginal ice zone and floe breakups, four separate drifts were conducted. Here we analyse data from the first two drifts covering the periods 19 Jan – 22 Feb and 24 Feb – 20 March 2015. These distinct drift periods are highlighted in Figs 1–3, 5 and 7. For further details of the field campaign please refer to Granskog *et al*.^33^.

### Meteorological observations

The N-ICE2015 meteorological observations^81^ were first presented by Cohen *et al*.^34^. Wind speed and direction were measured on a 10 m mast at the ice camp, located approximately 300 m from the ship, using a 2D ultrasonic wind sensor (Lufft Ventus V200A- UMB). Mean sea level pressure was measured with an RM Young 61302 V pressure sensor at the base of the mast. The 2 m air temperature was measured with a Vaisala HMP155 sensor mounted inside a triple-walled actively aspirated radiation shield (RM Young Model 43502). We averaged these processed data from 10-minute resolution to six-hour means (Fig. 2). Precipitation was collected in a Tretyakov shielded gauge, mounted 1–1.5 m above the surface and located 10 to 15 m from the meteorological tower. The contents of the gauge were collected daily at ~0700 UTC, melted, and the water equivalent amount was measured to the nearest millimetre. Wind–under-catch loss is estimated to be on the order of 25 to 30% and can be up to 50% for high wind speeds^82^ (>10 m s^−1^). Hence, the precipitation measurements are likely to be biased low due to blowing snow, especially during storm periods. For further details about these datasets, quality control, and uncertainties, refer to Cohen *et al*.^34^. For comparison with the precipitation observations, we also present the total daily precipitation from the European Centre for Medium Range Weather Forecasting’s Interim Re-Analysis (ERA-Interim)^83^ at the ice camp position. The timing of precipitation events in the Arctic is known to be very consistent among reanalyses, although there is a spread in the absolute values of total precipitation^27^. Note that we use the total precipitation product and not the snowfall. This is because a significant fraction of the total precipitation in the central Arctic is classified as rain, even during midwinter and sub-zero temperatures in ERA-I^27,46^. Downward longwave radiative fluxes^84^ were first presented by Walden *et al*.^37^. These were measured using a heated and ventilated Kipp and Zonen CGR4 pyrgeometer^37^. We averaged the processed data files from 1-minute resolution to six-hour means (Fig. 2). The atmospheric moisture content was calculated by Kayser *et al*.^36^, using balloon borne data^85^. These radiosondes (Vaisala RS92) were launched from the ice camp daily at 1100 and 2300 UTC^36^.

### Definition of a storm

The storm periods highlighted in Figs 1, 2, 3, 5 and 7 were first given by Cohen *et al*.^34^. These storms (M1 – M6) were defined locally at the N-ICE2015 camp, as periods with a rapid pressure drop of at least 5 hPa/6 hrs and sustained wind speeds of more than 7 m s^−1^ (Fig. 2). In addition, we show the tracks of storms M1 – M6 in Figs S1–S2, classified by The University of Melbourne’s cyclone identification scheme^35^ using ERA-Interim reanalysis^83^.

### Buoy observations

Several Sea Ice Mass Balance buoys (SIMBA), developed by the Scottish Association for Marine Research^86^, were deployed between 15–29 January as part of N-ICE2015^38,54,87^. The buoys were deployed on level ice and consisted of a 5 m long thermistor chain hanging through the air-snow-ice-ocean. The chain was comprised of solid state sensors that measured temperature with an accuracy of 0.1 °C at 2 cm vertical resolution every six hours. In addition, the sensors had a heating mode that recorded a proxy for thermal diffusivity. In this study we use data^88^ from two buoys, SIMBA_2015a and SIMBA_2015f (Fig. 3). The interfaces^89^ between ice-water, ice-snow and snow-air, the flooding of snow, and the ocean heat fluxes^89^ were determined by Provost *et al*.^38^. We smooth all times series using a 36-h running mean.

The ice drift speed in Fig. 3a was calculated using the GPS position of SIMBA_2015a^88^. Itkin *et al*.^54^ calculated the mean ice drift speed in Fig. 5a using hourly GPS positions from 16 buoys (combination of SIMBAs and simple GPS drifters) deployed at locations within a radius of 120 km around the N-ICE2015 ice camp^88^.

### Idealized model simulations

We apply the 1-D thermodynamic sea ice model, HIGHTSI^90^, for a case study of N-ICE2015, covering the period September 2014 – March 2015. The design of the experiment is similar to the set up described by Merkouriadi *et al*.^46^. Where available, we force the model with N-ICE2015 observations. At other times, we use the ERA-Interim reanalysis^83^. An earlier comprehensive evaluation of ERA-Interim for the N-ICE2015 winter found good agreement between the reanalysis and observations^5^. For the period before the field campaign we apply the reanalysis along the satellite derived back trajectory^54^ of the ice floe from Drift 1. We apply a constant ocean heat flux of 2.7 W m^−2^ throughout the simulation (a mean value suggested by McPhee *et al*.^91^). We prescribe an initial ice thickness on 1^st^ September of 1.2 m, and snow depth of 0 cm. The initial ice temperature is assumed to decrease linearly from −0.25 °C at the ice top to −1.9 °C at the ice bottom. For further details of the experiment set up see Merkouriadi *et al*.^46^.

We conduct two idealised experiments to explore the impact of changes in precipitation on sea ice mass balance (Fig. 4). In the first simulation we use the total precipitation from ERA-Interim (1*Precip). In the next simulation we reduce precipitation by 50% (0.5*Precip). This simple case study only considers changes in the magnitude of precipitation. We do not explore how changes in the timing of storms (and thus precipitation events) during winter impact on ice growth. Further sensitivity experiments were conducted where the initial ice thickness was varied from 1.0–1.5 m and where the initial ice temperature profile was altered. We also explored different oceanic heat fluxes^46,91^. These changes did not alter any conclusions drawn.

### Sea ice concentration at ice camp

Fransson *et al*.^57^ derived the mean daily sea ice concentration around the ice camp from the AMSR2 microwave radiometer^92^ (Fig. 5b). These data have a spatial resolution of 6.25 × 6.25 km^2^. The ice concentration was averaged over a 40 km square (7 × 7 grid cells), centred on the camp^57^.

### Sea ice classifications from satellite remote sensing, sea ice thickness and new ice volume estimates

We use TerraSAR-X (TSX, © DLR 2015) synthetic aperture radar images to calculate the lead fraction around the N-ICE2015 camp. We process the images to 40 meters ground resolution and segment these using the method from Doulgeris and Eltoft^93^ and Doulgeris^94^ (Fig. 6). We subsequently classified the segments into two classes, using auxiliary information. The two classes are defined as leads (open water and thin ice) and the remaining ice cover (level and deformed ice).

We estimate the volume of new ice growth in leads opened by storms using the areal fraction of leads calculated in the segmented images, as well as ice thickness measurements taken within these leads on 12 and 14 February (on average 0.28 m thick). Lead ice thickness surveys were conducted by drilling. We assume an average sea ice thickness of existing snow-covered ice of 1.0–1.5 m, based on extensive ice thickness measurements from Rösel *et al*.^42^. Rösel *et al*.^42^ measured the total thickness of snow and sea ice by electro-magnetic induction (EM31SH, Geonics Ltd.) on a large scale. From this, they calculated the ice thickness by subtracting the snow depth measured with a snow probe (Magnaprobe, Snow-Hydro)^42^.

### Oceanographic observations

Ocean-to-ice heat fluxes^95^ shown in Fig. 5 were measured with a Turbulent Instrument Cluster (TIC) installed at a depth of 1 m below the ice surface during Drift 1 and during a short period of Drift 2. The TIC was located a few hundred meters from the ship to avoid sampling in its wake. The TIC was equipped with a Sontek Acoustic Doppler Velocimeter (ADV), sampling at 24 Hz, to measure ocean currents. In addition, there were Sea-Bird Electronics sensors, SBE3F and SBE4, to measure temperature and conductivity at 24 Hz. Processing of the TIC data and calculation of ocean heat fluxes at the ice-ocean interface was performed by Peterson *et al*.^59^, and followed standard methods reported by McPhee *et al*.^91^.

We calculate the mean ocean-ice heat flux (from 1 m TIC) during the winter storms to be 6.7 W m^−2^. Storm periods are based on Cohen *et al*.^34^, and are highlighted in Fig. 5. The mean ocean-ice heat flux (from 1 m TIC) during the winter period when there is no forcing (i.e. no storms, no steep topography below, and no shallow Atlantic Water) is 2.2 W m^−2^. Mixing in the ocean is often still enhanced a few days after a storm (Fig. 5). Therefore, we define ‘no storm’ periods as the time between 3 days after the end of a storm and the day before the start of the next storm, to avoid any contamination.

Dissipation rates, mixed layer depth, and ocean heat fluxes at the pycnocline depth for the N-ICE2015 winter, shown in Fig. 5, were calculated by Meyer *et al*.^60^ using microstructure profile observations^96^. Typically, 3 sets of 3 microstructure profiles were conducted daily between 0800 UTC and 2000 UTC, using two loosely tethered free-fall MSS-90 microstructure profilers^97^ developed by ISW Wassermesstechnik. Each final set of microstructure profiles is therefore the result of 3 combined individual microstructure profiles. These sets were used to estimate the profiles of ocean heat fluxes presented in Fig. S4. The MSS-90 microstructure profilers contained precision conductivity, temperature, pressure, and microstructure sensors and sampled at 1024 Hz. Profiles were made through a hole in the ice adjacent to the TIC installation. Data processing followed the method of Fer *et al*.^98^. The processed data provide 0.2 m averaged temperature and salinity measurements and 1 m averaged dissipation rates. The mixed layer depth shown in Fig. 5e was calculated by Meyer *et al*.^62^.

The *f*CO2 observations^99^ shown in Fig. 5 were processed by Fransson *et al*.^57^. The data were obtained by infrared analysis of equilibrator headspace samples. The seawater was supplied from an intake located midships, at approximately 5 m water depth. The measurements were made using an instrument supplied by General Oceanics and designed following the principles presented by Pierrot *et al*.^100^ using two-stage showerhead equilibration and a LICOR 7000 nondispersive infrared detector^57^.

### Large-scale impact of storms

To investigate the large scale impact of the six N-ICE2015 winter storms, we use satellite sea ice concentration and drift vectors from the Ocean and Sea Ice Satellite Application Facility (OSI-SAF) and the ERA-Interim atmospheric reanalysis^83^. Specifically, we show the sea ice extent before and after each of the six N-ICE2015 storms. Together with this we show the 10 m wind speed and mean sea level pressure field averaged over the duration of each storm (Fig. 7). The direction of surface winds is typically aligned parallel to the mean sea level pressure isobars, and the flow is anti-clockwise around the low pressure centre, marked with an (L).

Using the OSI-SAF sea ice drift product with a 62.5 km resolution, we calculate the import/export of sea ice area into a box covering the Atlantic Water inflow region north of Svalbard, throughout the N-ICE2015 winter (Fig. 7). We define our Atlantic Water box on the OSI-SAF drift vector grid. The four corner coordinates are: 5.19°E, 82.80°N; 18.43°E, 79.71°N; 39.29°E, 80.74°N; 35.54°E, 84.39°N. To calculate the net ice area import or export within the box, we multiply the ice drift velocity from OSI-SAF perpendicular to the box edge with the sea ice concentration at that grid point and the grid length. We sum this total around the perimeter of the box. Using the OSI-SAF sea ice concentration product, we calculate the daily change in sea ice area within the box. Note, the time period for averaging over the storms in Fig. 7a–f is chosen to coincide with the reduction in mean sea ice concentration within this box (Fig. 7g). From the daily change in ice concentration, we subtract the area of ice import/export to the box. Finally, we make the assumption that the remaining area loss of sea ice is caused by ice melt, due to storm driven mixing of warm Atlantic Water. We calculate the required ocean heat flux to melt this ice, assuming three different constant ice thicknesses to the sea ice area (1.00 m, 1.25 m and 1.50 m). We use a constant ice density of 900 kg m^−3^, and use the specific latent heat of fusion of pure ice of 333500 J kg^−1^ multiplied by 0.89, following Bitz and Lipscomb^101^.

Caution should be taken with these heat flux estimates, because we do not account for ice lost due to convergence (e.g. ridging or rafting). However, we expect this term to be small compared to the ice-melt term. For comparison, ice export from the box contributed a maximum of 25% of the total area loss. Our method also does not account the melt of ice floes that only partially melt from below (e.g. Fig. 3b), since this is not detected in ice concentration measurements. The uncertainty of the ice concentration data for low ice concentrations in the marginal ice zone can exceed 10%^102^; this adds further uncertainty to the ice-melt estimate.

## Supplementary information

Supplementary Info

## Data Availability

All of the N-ICE2015 datasets used in this study are cited in the Methods section and publicly available through the Norwegian Polar Institute’s data centre (data.npolar.no), except for data in Fig. 3 that is available from the SEANOE data portal (seanoe.org). ERA-Interim files were downloaded from the University of Colorado Research Data Archive (rda.ucar.edu). OSI-SAF sea ice concentration and ice drift data were downloaded from the EUMETSAT Ocean and Sea Ice Satellite Application Facility (osisaf.met.no). AMSR-2 sea ice concentration was downloaded from University of Bremen (seaice.uni-bremen.de), and NSIDC sea ice extent from the National Snow and Ice Data Center (nsidc.org). TerraSAR-X images were provided by the German Aerospace Center (DLR) through TerraSAR-X AO OCE2582. Storm tracks shown in Figures S1 and S2 were provided by Irina Rudeva and Ian Simmonds at the University of Melbourne.
