# Supplementary material for: Winter storms accelerate the demise of sea ice in the Atlantic sector of the Arctic Ocean
Source: Sci Rep. 2019 Jun 25;9:9222. doi: 10.1038/s41598-019-45574-5 (PMC6592951; doi:10.1038/s41598-019-45574-5)
Supplement: Supplementary file 1 — Supplementary Info [file 41598_2019_45574_MOESM1_ESM.pdf]

Winter storms accelerate the demise of sea ice in the Atlantic Sector of the Arctic Ocean

Robert M. Graham[1*]†, Polona Itkin[1*]†, Amelie Meyer[1-2], Arild Sundfjord[1], Gunnar Spreen[3], Lars H. Smedsrud[4-5], Glen E. Liston[6], Bin Cheng[7], Lana Cohen[1], Dmitry Divine[1], Ilker Fer[4-5], Agneta Fransson[1], Sebastian Gerland[1], Jari Haapala[7], Stephen R. Hudson[1], A. Malin Johansson[8], Jennifer King[1], Ioanna Merkouriadi[1], Algot K. Peterson[4-5], Christine Provost[9], Achim Randelhoff[1], Annette Rinke[10], Anja Rösel[1], Nathalie Sennéchael[9], Von P. Walden[11], Pedro Duarte[1], Philipp Assmy[1], Harald Steen[1], and Mats A. Granskog[1*]

†Contributed equally to this study
*Corresponding authors:
Mats.Granskog@npolar.no, Polona.Itkin@gmail.com, Robert.Graham@npolar.no

Submitted to Scientific Reports: 10 December 2018

Revised: 29 May 2019

Affiliations

1. Norwegian Polar Institute, Fram Centre, Tromsø, Norway
2. ARC Centre of Excellence for Climate Extremes, IMAS University of Tasmania, Hobart, Australia
3. Institute of Environmental Physics, University of Bremen, Bremen, Germany
4. Geophysical Institute, University of Bergen, Bergen, Norway
5. Bjerknes Centre for Climate Research, Bergen, Norway
6. Cooperative Institute for Research in the Atmosphere, Colorado State University, Fort Collins, Colorado, USA
7. Finnish Meteorological Institute, Helsinki, Finland
8. UiT The Arctic University of Norway, Tromsø, Norway
9. Laboratoire LOCEAN-IPSL, Sorbonne Universités, UPMC, Univ. Paris 6, CNRS-IRD-MNHN, Paris, France
10. Alfred Wegener Institute, Helmholtz Centre for Polar and Marine Research, Potsdam, Germany
11. Washington State University, Department of Civil and Environmental Engineering, Pullman, Washington, USA

Supplementary Materials

Fig. S1. Map of all storm tracks
Fig. S2. Synoptic maps for Phase-I and Phase-II of storms M1-M6
Fig. S3. Mechanism of flooding and snow-ice formation.
Fig. S4. Vertical profiles of ocean heat fluxes from microstructure profiler observations.

S1: Storm tracks

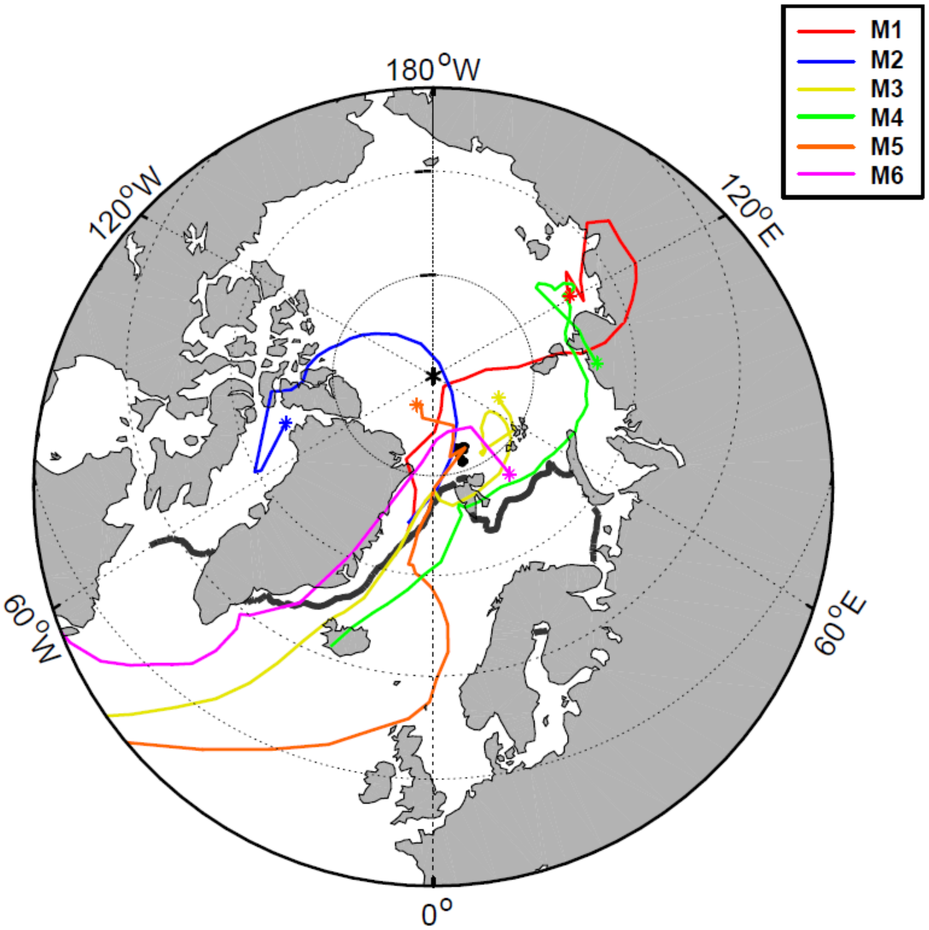

Fig S1. Map of storm tracks for Storms M1-M6, *based on The University of Melbourne cyclone identification scheme[35] applied to ERA-Interim[83]. Storms tracks are shown in colour (see legend). The end of the storm track is marked by a star. Mean sea ice extent from January-March 2015 (15%, ERA-Interim) is given by thick black line. N-ICE2015 Drift 1 and Drift 2 are shown in black.*

S2: Synoptic maps for Phase-I and Phase-II of the six N-ICE2015 winter storms

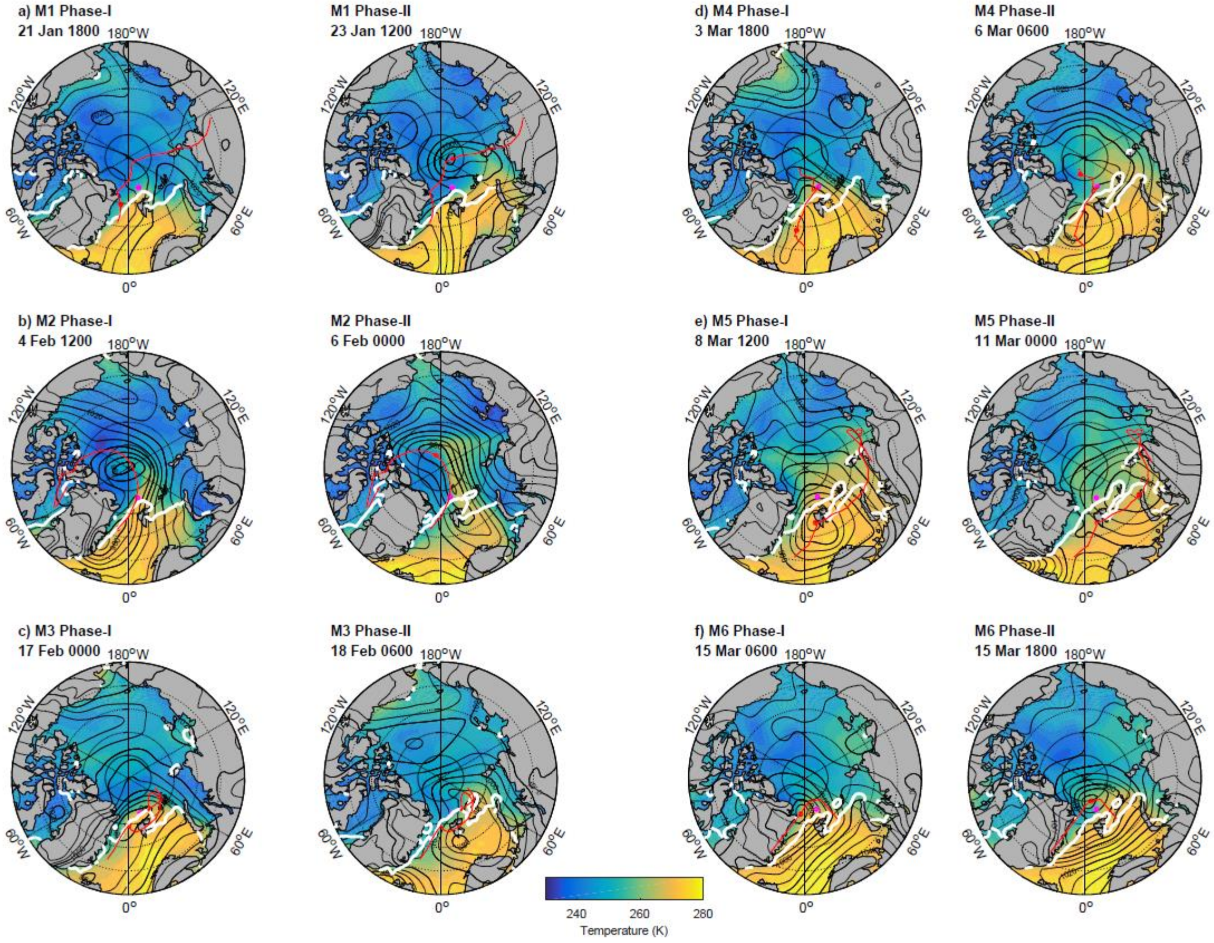

Fig S2**. Maps show snap shots during Phase-I and Phase-II of storms M1-M6. Maps of mean sea level pressure (black contours, [hPa]), 2 m temperature (colour bar, [K]), and sea ice extent (white contour, [15%]) from ERA-Interim.* *Date and valid time for the synoptic chart are given below the name of each storm. The position of the N-ICE2015 camp at this time is given by a pink dot. The track of the given storm is shown by the red line (see* ***Fig S1*), and the centre of the storm at the valid time is given by a red dot. Winds blow anti-clockwise around this low pressure centre. Phase-I of a storm corresponds to the time before the storm's low pressure centre passes the N-ICE2015 camp. Phase-II corresponds to the time after the low pressure centre has crossed the camp. Typically Phase-I is characterised by strong southerly winds, and warm and moist air advection. In contrast, Phase-II is characterised by northerly winds and cold, dry air advection.*

S3: Mechanism of flooding and snow-ice formation

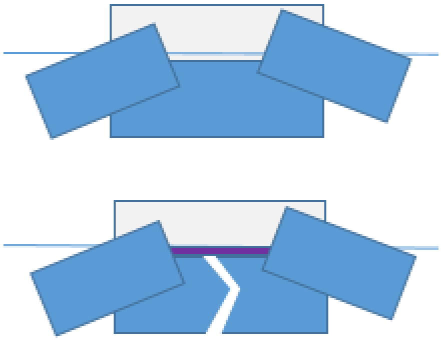

***Fig S3. Schematic of mechanism behind snow-ice formation after storms**. Thin blue line delineates the water surface and the purple layer on the bottom panel is the flooded snow that will freeze into snow-ice when the temperatures sink to and below the freezing point after the winter storm passes. The pathway for water to flood the bottom of the snowpack is created by cracks in the ice floe caused by deformation during storms. This likely happens e.g. in case of the buoy in **Fig 3c**.*

S4 Ocean heat fluxes from microstructure profiles

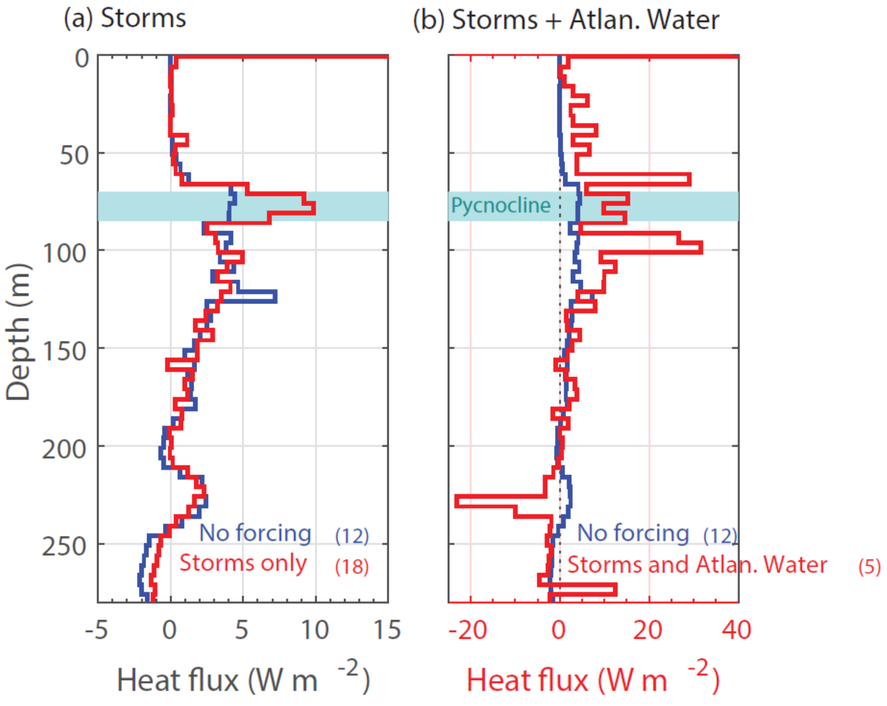

***Fig S4**: **Mean vertical profiles of heat fluxes during the winter period of the N-ICE2015** campaign sampled with a microstructure profiler. We subsample the profile sets for 'no forcing' conditions (blue profiles), and for sets affected by (a) storms and (b) storms and Atlantic Water combined. 'No forcing' means no storms, no shallow Atlantic Water, and no steep topography. Sets of profiles used to make the mean profiles for a given forcing exclude sets where other forcings are present. The number of sets of profiles used to make the mean profiles are given in brackets. The winter microstructure profiler data set consists of 46 sets of mixing and heat flux vertical profiles with 1 m vertical resolution as described in Methods. Note the different x-axis scale on subplots - for comparison, the blue 'no forcing' profiles are identical.*
